# Supplementary material for: Female sexual behavior in mice is controlled by kisspeptin neurons
Source: Nat Commun. 2018 Jan 26;9:400. doi: 10.1038/s41467-017-02797-2 (PMC5786055; doi:10.1038/s41467-017-02797-2)
Supplement: Supplementary file 1 — Supplementary Information [file 41467_2017_2797_MOESM1_ESM.pdf]

Supplemental Information

Female sexual behavior in mice is controlled by kisspeptin neurons

Hellier et al.

Supplementary Figures

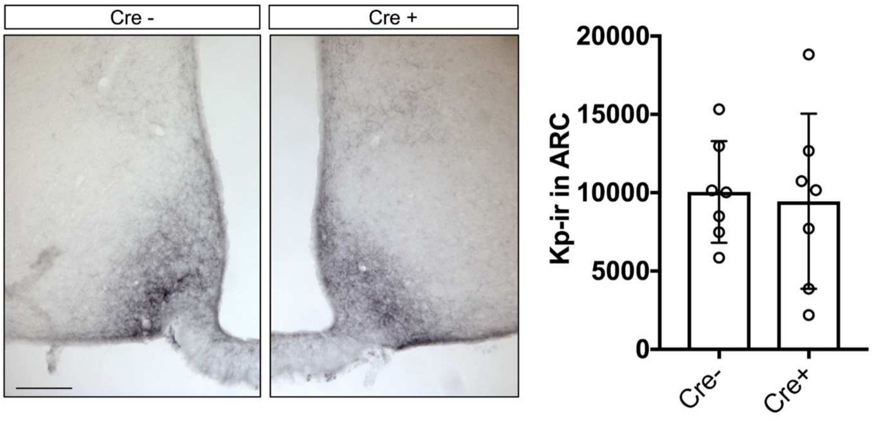

Supplementary Fig. 1: Kisspeptin expression in the arcuate nucleus is unaffected by viral ablation of the RP3V kisspeptin population. (a) Photomicrographs showing examples of kisspeptin-immunoreactivity in a *KissIC* (*Cre*$^{-}$, left) and *KissIC* (*Cre*$^{+}$, right) mouse; (b) Total amount of kisspeptin (Kp)-immunoreactivity indicated as total area (µm^2) covered by thresholded pixels. Unpaired two-tailed t test; *P*=0.81; n=7 per genotype; Scale bar represents 100 µm. Bars represent means ± SEM. For all experimental details, see supplementary Table 1.

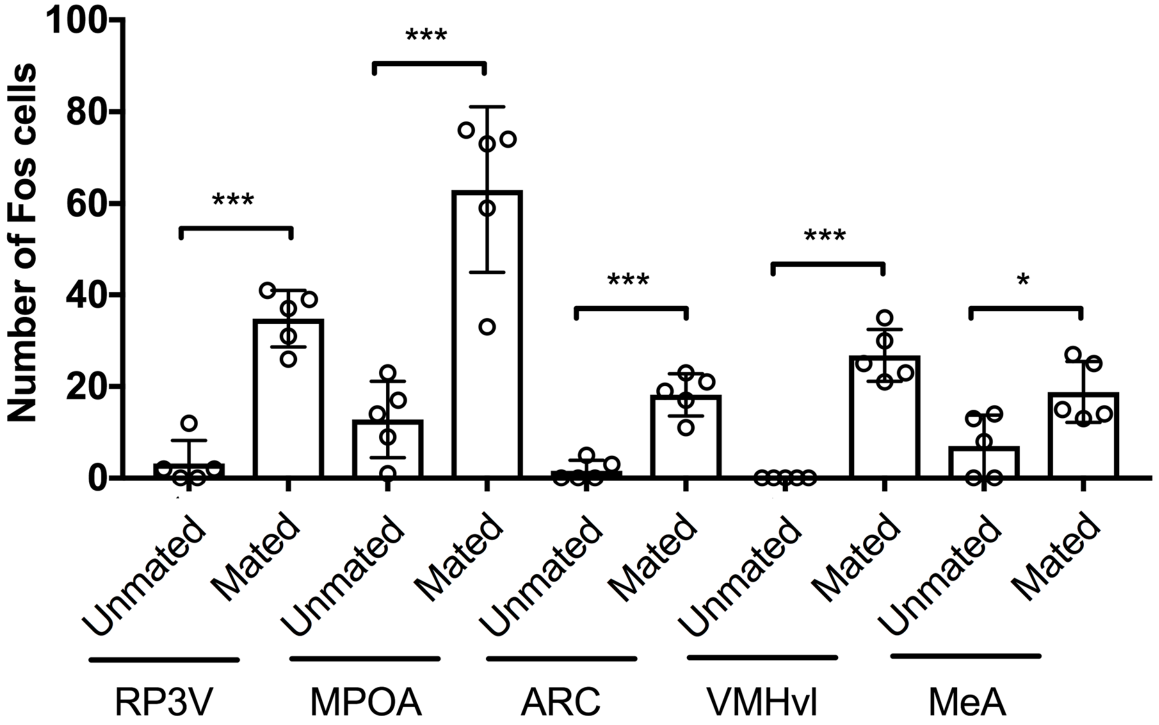

Supplementary Fig. 2: Fos activation upon mating in hypothalamic regions implicated in sexual behavior. Ovary intact, female mice were either mated with a sexually active male for 15 min or left alone in their homecage. Brains were processed for Fos immunoreactivity. Abbreviations are as follows: RP3V rostral periventricular area of the third ventricle of the hypothalamus; MPOA, medial preoptic area; ARC, arcuate nucleus; VMHvl, ventrolateral part of the ventromedial hypothalamus; MeA, medial amygdala. Bars represent means ± SEM. Tukey's multiple comparison test; *$P \leq 0.05$, ***$P \leq 0.001$ compared to unmated; n=5. For all experimental details, see supplementary Table 1.

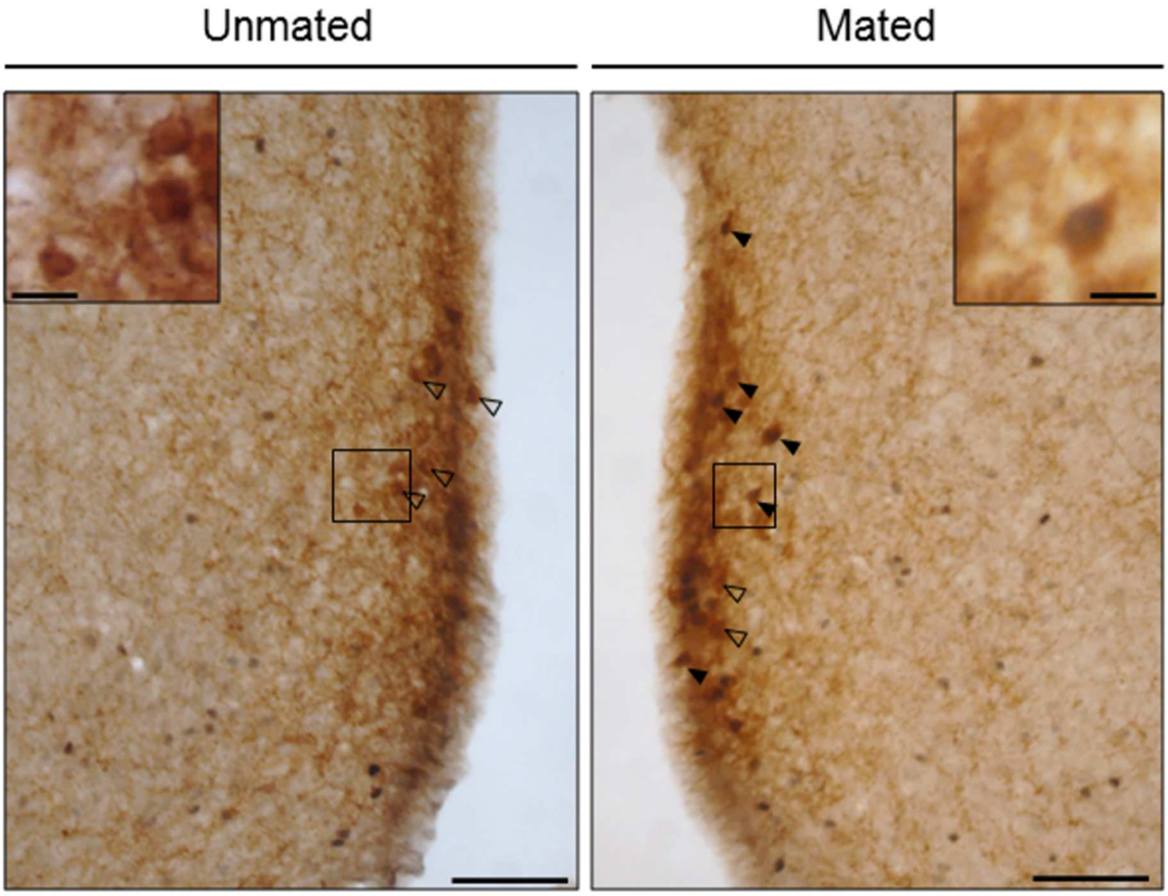

Supplementary Fig. 3: Fos/kisspeptin double-labeling upon mating in the RP3V. Representative photomicrographs from an unmated female (left panel) and mated female (right panel). Inserts show higher magnification. Black arrow heads show double-labeled Fos (in blue)/kisspeptin (in brown) neurons. White arrow heads show single-labeled kisspeptin neurons detected in brown. Scale bar represents 100 µm and 10 µm, respectively. For all experimental details, see supplementary Table 1.

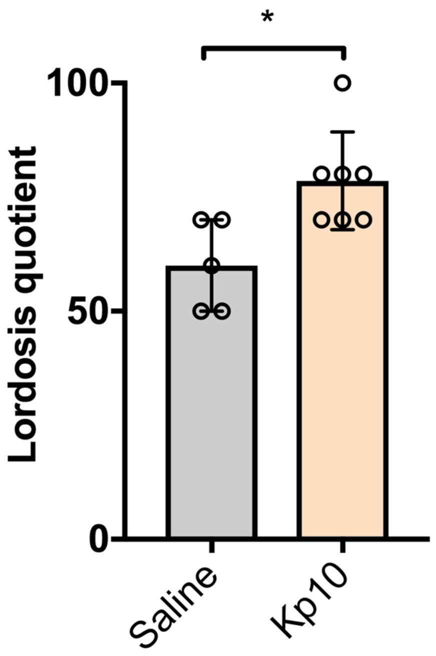

Supplementary Fig. 4: An intracerebroventricular injection with kisspeptin (Kp-10; 10.4 ng/kg) stimulates lordosis behavior in *WT* female mice. Bars represent means ± SEM and the number of animals for each experimental group is given in each bar. Unpaired t test; *$P \leq 0.05$; n=5 (saline) and 7 (Kp10). For all experimental details, see supplementary Table 1.

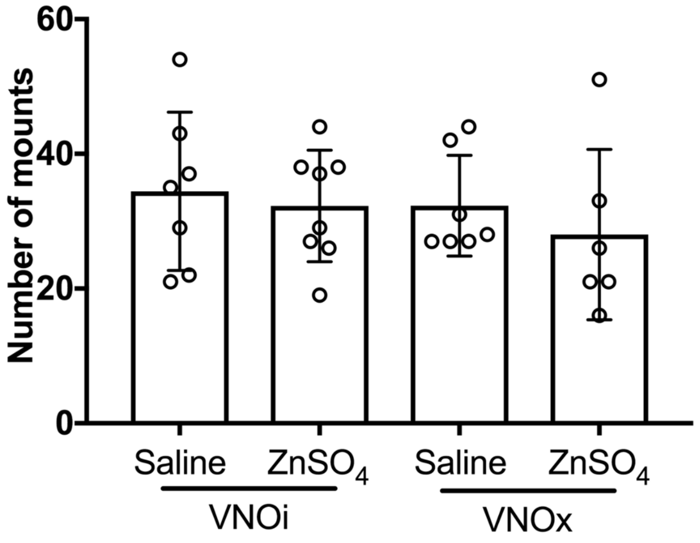

Supplementary Fig. 5: VNO removal (VNOx) or ablation of the MOE by intranasal infusion with a zinc sulfate ($ZnSO_4$) solution did not affect the number of mounts received from the stimulus male. Bars represent means ± SEM. Dunn's multiple comparison test; P>0.99 for each group compared to Saline/VNOi group; n=7/8/7/6. For all experimental details, see supplementary Table 1.

Supplementary Table 1. Genetic background of the mouse models used and hormonal treatments before the behavioral tests.

	Mouse model			Treatments						N
	Genotype	Genetic Background	Surgeries	Viruses*	E_2 (sc)	Prog (sc) **	Treatment	Injection	Stimulation	
**Fig. 1**										
a	*WT*	*C57Bl/6J*	OVX / VNOx	-	x	x	Saline / $ZnSO_4$	intranasal	Clean or Male-soiled bedding	28/9/7/8/9
b	$Kiss^{+/+}$ & $Kiss^{-/-}$	*129/SvJ*	OVX	-	x	x	Kp-10	sc	Male and female odors	9 per genotype
c-d	$KissCre^{-/-}$ & $KissCre^{+/+}$	*Mixed C57Bl/6J & 129/SvJ*	OVX	*AAV-flex-taCasp3-TEVp*	x	x	-	-	Mating	7 per genotype
e	$KissCre^{-/-}$ & $KissCre^{+/+}$	*Mixed C57Bl/6J & 129/SvJ*	OVX	*AAV-flex-taCasp3-TEVp*	x	x	- / Kp10	sc	Male and female odors	7 per genotype
**Fig. 2**										
a	*WT*	*C57Bl/6J*	-	-	-	-	-	-	Mating	3 (Unmated) / 5 (Mated)
b	*WT*	*C57Bl/6J*	OVX	-	x	-	Saline/ Kp10	sc	Mating	8 per group
c-d	$Kiss^{+/+}$ & $Kiss^{-/-}$	*129/SvJ*	OVX	-	x	x	- / Kp10	sc	Mating	7 (Kiss+/+) /

										10 (Kiss-/-)
e-f	*KissCre*$^{-/-}$ & *KissCre*$^{+/+}$	*Mixed C57Bl/6J & 129/SvJ*	OVX	*AAV-flex-taCasp3-TEVp*	x	x	- / Kp10	sc	Mating	7 per genotype
I	*KissCre*$^{-/-}$ & *KissCre*$^{+/+}$	*Mixed C57Bl/6J & 129/SvJ*	OVX	*AAV5-EF1a-DIO-hChR2(H134R)-mCherry-WPRE-pA*	x	-	Blue light***		Mating	8 per genotype
	**Fig. 3**									
a	*WT*	*C57Bl/6J*	OVX / VNOx	-	x	x	Saline / ZnSO4	intranasal	Mating	8/9/8/7
b	*WT*	*C57Bl/6J*	OVX / VNOx	-	x	x	Saline / ZnSO4	intranasal	Mating	28/8/9/8/7
	**Fig. 4**									
**a**	*Dicer*$^{loxP/loxP}$ & *GnRH::Cre;Dicer*$^{loxP/loxP}$	*C57Bl/6J*	OVX	-	x	x	Saline/Kp10	sc	Male and female odors	6 Dicer$^{loxP/loxP}$ & 8 GnRH::Cre;Dicer$^{loxP/loxP}$
**a**	*Dicer*$^{loxP/loxP}$ & *GnRH::Cre;Dicer*$^{loxP/loxP}$	*C57Bl/6J*	OVX	-	x	-	GnRH	sc	Male and female odors	6 Dicer$^{loxP/loxP}$ & 8 GnRH::Cre;Dicer$^{loxP/loxP}$

**b**	*Dicer*$^{loxP/loxP}$ & *GnRH::Cre;Dicer*$^{loxP/loxP}$	*C57Bl/6J*	OVX	-	x	x	-		Mating	7/8
**c**	*Kiss*$^{+/+}$ & *Kiss*$^{-/-}$	*129/SvJ*	OVX	-	x	-	- / GnRH	sc	Mating	10 per treatment
	**Fig. 5**									
**a**	*KissIC/R26-BlZ*	*Mixed C57Bl/6J & 129/SvJ*	-	*AAV5-EF1a-DIO-hChR2(H134R)-mCherry-WPRE-pA*	-	-	-	-	-	3 (unknown estrous cycle stage)
**b-h**	*KissIC/R26-BlZ*	*Mixed C57Bl/6J & 129/SvJ*	-	-	-	-	-	-	-	6 (proestrus) and 8 (metestrus/ diestrus)
	**Fig. 6**									
**a**	*nNOS*$^{+/+}$ & *nNOS*$^{-/-}$	*C57Bl/6J*	OVX	-	x	x	Saline / SNAP	sc	Male and female odors	6 (nNOS$^{+/+}$) / 7 (nNOS$^{-/-}$)
**b**	*nNOS*$^{+/+}$ & *nNOS*$^{-/-}$	*C57Bl/6J*	OVX	-	x	x	Saline/ SNAP	sc	Mating	7 per group
**c**	*nNOS*$^{-/-}$	*C57Bl/6J*	OVX	-	x	x	- / Kp10	sc	Mating	7 per group
**c**	*nNOS*$^{-/-}$	*C57Bl/6J*	OVX	-	x	-	GnRH	sc	Mating	7 per treatment

**d**	*Kiss*$^{-/-}$	*129/SvJ*	OVX	-	x	x	- / SNAP	sc	Mating	10 per treatment
	**Supplementary Figs.**									
**1**	*KissCre*$^{-/-}$ & *KissCre*$^{+/+}$	*Mixed C57Bl/6J & 129/SvJ*	OVX	*AAV-flex-taCasp3-TEVp*	x	-	-	-	-	7 per genotype
**2-3**	*WT*	*C57Bl/6J*	-	-	-	-	-	-	Mating	5 per group
**4**	*WT*	*C57Bl/6J*	OVX	-	x	x	Saline/ Kp10	icv	Mating	5/7
**5**	*WT*	*C57Bl/6J*	OVX	-	x	x	-	sc	Mating	7/8/7/6

*Viruses were injected bilaterally into the RP3V.
** Progesterone was administered 3h prior to behavioral experiments.
***Photostimulation at 10 Hz, 473 nm.

All experimental females were brought into behavioral estrus by adult ovariectomy and combined treatment with estradiol and progesterone unless stated otherwise. Furthermore, levels of female sexual behavior displayed by the control (wild-type) females vary as function of the background strain with *129/SvJ* females showing relatively low levels compared to *C57Bl/6J* females. Abbreviations: WT, wild-type; E2, estradiol; P, progesterone; sc, subcutaneous; icv, intracerebroventricular; Kp10, kisspeptin; SNAP, S-nitroso-N-acetylpenicillamine; RP3V, rostral periventricular area of the third ventricle of the hypothalamus.

Supplementary Table 2. Reagents and resources used in the present study.

REAGENT or RESOURCE	SOURCE	IDENTIFIER
Antibodies		
Rabbit polyclonal anti-cFos	Santa Cruz	Cat# Sc-52R<br>AB_2106783
Living Colors® rabbit polyclonal anti-ds-red	Clontech	Cat# 9632496
Goat polyclonal anti-wheat germ agglutinin (WGA)	Vector Laboratories	Cat# AS-2024<br>AB_2315608
Horse polyclonal biotinylated anti-goat	Vector Laboratories	Cat# BA-9500<br>AB_2336123
Cy3®-conjugated AffiniPure Fab Fragment donkey anti-rabbit IgG	Jackson ImmunoResearch	Cat# 711-167-003<br>AB_2340606
Rabbit polyclonal anti-kisspeptin 10	Millipore	Cat# AB9754<br>AB_2296529
Goat polyclonal biotinylated anti-rabbit	Dako	Cat# B0432
Rabbit polyclonal anti-nNOS	Life technologies	Cat# 61-7000<br>AB_88207

Bacterial and Virus Strains		
AAV5-flex-taCasp3-TEVp	University of North Carolina Vector Core [23]	N/A
AAV5-EF1a-DIO-hChR2(H134R)-mCherry-WPRE-pA	University of North Carolina Vector Core [62]	N/A
AAV9-EF1a-DIO-hChR2-(H134R)-mCherry-WPRE-hGH	Penn Vector Core [62]	N/A
Chemicals, Peptides, and Recombinant Proteins		
17β-estradiol	Sigma	E8875
Progesterone	Sigma	P0130
Mouse kisspeptin 10 (Kp-10)	NeoMPS, Strassbourg, France	N/A
Gonadotropin-releasing hormone (GnRH)	Polypeptide Laboratories France SAS	SC087
S-nitroso-N-acetyl-DL-penicillamine (SNAP)	Sigma	N398
BAY41-2272	Sigma	B8810
Streptavidin-conjugated Cy5®	Invitrogen	SA-1011

Critical Commercial Assays		
Tyramide Signal Amplification Plus (TSA+) biotin kit	PerkinElmer	NEL749A001KT
Avidin-biotin complex (ABC) kit	Vector Laboratories	PK-6100
Experimental Models: Organisms/Strains		
Mouse: Kisspeptin knockout (*Kiss*$^{-/-}$)	[22]	N/A
Mouse: Kisspeptin-IRES-Cre (*KissIC*)	[26]	N/A
Mouse: GPR54-IRES-Cre (*GPIC*)	[29]	N/A
Mouse: *GnRH::Cre*	[12]	N/A
Mouse: *Dicer*$^{loxP/loxP}$	[59]	N/A
Mouse: *R26-BIZ*	[31]	N/A
Mouse: nNOS knockout (*nNOS*$^{-/-}$)	[54, 60]	N/A
